# The impact of robustness of deformable image registration on contour propagation and dose accumulation for head and neck adaptive radiotherapy

**DOI:** 10.1002/acm2.12361

**Published:** 2018-05-30

**Authors:** Lian Zhang, Zhi Wang, Chengyu Shi, Tengfei Long, X. George Xu

**Affiliations:** ^1^ Center of Radiological Medical Physics University of Science and Technology of China Hefei Anhui Province China; ^2^ Department of Radiation Oncology The First Affiliated Hospital of Anhui Medical University Hefei Anhui Province China; ^3^ Department of Medical Physics Memorial Sloan‐Kettering Cancer Center New York NY USA; ^4^ Nuclear Engineering Program Rensselaer Polytechnic Institute Troy NY USA

**Keywords:** adaptive radiotherapy, contour propagation, deformable image registration, dose accumulation

## Abstract

Deformable image registration (DIR) is the key process for contour propagation and dose accumulation in adaptive radiation therapy (ART). However, currently, ART suffers from a lack of understanding of “robustness” of the process involving the image contour based on DIR and subsequent dose variations caused by algorithm itself and the presetting parameters. The purpose of this research is to evaluate the DIR caused variations for contour propagation and dose accumulation during ART using the RayStation treatment planning system. Ten head and neck cancer patients were selected for retrospective studies. Contours were performed by a single radiation oncologist and new treatment plans were generated on the weekly CT scans for all patients. For each DIR process, four deformation vector fields (DVFs) were generated to propagate contours and accumulate weekly dose by the following algorithms: (a) ANACONDA with simple presetting parameters, (b) ANACONDA with detailed presetting parameters, (c) MORFEUS with simple presetting parameters, and (d) MORFEUS with detailed presetting parameters. The geometric evaluation considered DICE coefficient and Hausdorff distance. The dosimetric evaluation included D_95_, D_max_, D_mean_, D_min_, and Homogeneity Index. For geometric evaluation, the DICE coefficient variations of the GTV were found to be 0.78 ± 0.11, 0.96 ± 0.02, 0.64 ± 0.15, and 0.91 ± 0.03 for simple ANACONDA, detailed ANACONDA, simple MORFEUS, and detailed MORFEUS, respectively. For dosimetric evaluation, the corresponding Homogeneity Index variations were found to be 0.137 ± 0.115, 0.006 ± 0.032, 0.197 ± 0.096, and 0.006 ± 0.033, respectively. The coherent geometric and dosimetric variations also consisted in large organs and small organs. Overall, the results demonstrated that the contour propagation and dose accumulation in clinical ART were influenced by the DIR algorithm, and to a greater extent by the presetting parameters. A quality assurance procedure should be established for the proper use of a commercial DIR for adaptive radiation therapy.

## 1 INTRODUCTION

Treatment of head and neck (H&N) cancers has been found to benefit from Intensity Modulated Radiation Therapy (IMRT).^1^, ^2^, ^3^ IMRT plans are typically based on the anatomy defined by a pretreatment CT image dataset of the patient. The desire to consider potential changes in patient anatomy during the treatment period — due to weight loss and tumor shrinkage — led to the new approach called adaptive radiotherapy (ART).^4^, ^5^, ^6^ In ART, the original treatment plans are revised to address the random and systematic patient anatomical variations over the 6–7 weeks of fractionated delivery. For H&N patients, weight loss and volume shrinkage lead to changes to the target and parotid glands, and the adaptive re‐planning has been shown to be successful in compensating for the geometrical changes.^7^, ^8^, ^9^, ^10^ However, off‐line ART involving manual delineation adjustment of the OARs and the target in H&N cases is a labor and time‐consuming procedure.^11^ More recent advances in the deformable image registration (DIR) further improved the efficiency of the ART workflow. DIR plays an important role in efficient adaptive treatment planning.^12^, ^13^, ^14^ DIR allows the propagation of contours as well as the corresponding radiation dose from one image to another.^15^ The ability to perform contour propagation facilitates an efficient adaptive radiotherapy workflow by avoiding tedious manual delineation.^16^ Dose mapping is used in treatment evaluation by accumulating the fractional or weekly dose during the treatment course through daily cone beam CTs (CBCTs) or CT‐on‐rails.^17^ In addition, DIR can be similarly used in 4D dose accumulation to study interplay effect and to map the densities from planning CT to CBCT in order to compute dose on the daily CBCT.^18^, ^19^

As more commercial treatment planning systems (TPSs) begin to integrate the DIR module in clinical adaptive radiotherapy, DIR‐associated uncertainty has drawn concerns due to its fundamental importance to contour propagation, dose accumulation, auto‐segmentation, and 4D‐CT processing.^20^, ^21^, ^22^ DIR algorithms are supposed to be well validated in both the TPS commissioning and routine quality assurance procedure. A limited number of publications have addressed the DIR validation research in H&N adaptive radiotherapy, especially for contour propagation and dose accumulation.^23^, ^24^, ^25^ In our work, adaptive radiotherapy was studied using CT‐on‐rails linac (CTVision, Siemens, Erlangen, Germany) and RayStation (Version 5.0.2, RaySearch Lab, Stockholm, Sweden).

The goal of this study is to evaluate two commercial DIR algorithms integrated in RayStation TPS for adaptive radiotherapy. Both geometric and dosimetric factors are considered in the evaluation to estimate the impact of DIR variation caused by the DIR itself and different presetting parameters, and also the influence of volume size. For comparison purpose, we used the simple presetting parameters and the detailed presetting parameters to initialize the DIR algorithms for adaptive radiotherapy. To our knowledge, this is the first time that a systematic evaluation is performed on the DIR algorithms of this commercial system clinically. Geometric and dosimetric evaluation coefficients were compared to show the variation caused by the DIR algorithm itself and different presetting parameters. This retrospective research focused on the robustness of the DIR algorithms integrated in RayStation when dealing with ART, and specially reported on the variations when handling contour propagation and dose accumulation under different conditions:

1. between the two commercial DIR algorithms by RayStation: ANACONDA vs MORFEUS;
2. between simple and detailed DIR presetting parameters for both algorithms;
3. between small and large organs for all the presettings and algorithms.

## 2 MATERIALS AND METHODS

### 2.A Patient data

In this study, ten H&N cancer patients were randomly selected. All the patients received off‐line adaptive treatment planning with several weekly CTs scanned using Siemens CT‐on‐rails during treatment fractions. The CT parameters were set to 3.0 mm thickness and 1.0 mm in plane resolution. For each weekly CT, CIVCO (Orange City, Iowa) H&N board and thermoplastic mask with a molded pillow were used to immobilize the patients. The target and organs at risk (OARs) contours were re‐delineated by the same radiation oncologist (see Table 1).

**Table 1:** The statistics of ten H&N cancer patients in this study

Patient no.	Age	Staging	Weekly GTV volume change (cm^3^)	Weekly patient weight loss (kg)
1	57	T3N2M0	−2.13 ± 0.47	−0.73 ± 0.22
2	44	T2N1M0	−0.76 ± 0.28	−0.44 ± 0.30
3	52	T3N2M0	−1.62 ± 0.66	−0.86 ± 0.51
4	61	T2N1M0	−0.70 ± 0.32	−0.48 ± 0.73
5	50	T3N2M0	−2.19 ± 0.81	−0.55 ± 0.65
6	51	T2N0M0	−0.04 ± 0.75	−0.74 ± 0.59
7	63	T2N1M0	−0.68 ± 0.76	−0.04 ± 0.80
8	41	T3N2M0	−1.98 ± 0.54	−0.35 ± 0.61
9	49	T2N2M0	−1.26 ± 0.57	−0.64 ± 0.37
10	47	T2N1M0	−0.26 ± 0.89	−0.10 ± 0.57

### 2.B Contour selection and treatment planning

For all the ten patients, contours including the GTV, left/right parotids, spinal cord, brainstem, left/right temporal lobes, left/right lens, left/right optic nerves, left/right cochleae were chosen as the reference structures. All the reference region‐of‐interest (ROI) contours were separated to two groups (large organs and small organs) with a threshold of 8 cm^3^ in volume size for better comparison, as discussed further in Section 2.B.^26^ Table 2 lists the volume statistics for all the reference ROIs. To exclude the uncertainties induced by the auto‐segmentation process, the reference contours on the primary and each weekly CTs were manually delineated by the same senior radiation oncologist (RO) on the RayStation TPS according to the RTOG guideline. The PTV was not selected as the reference contour because it can be created by expanding the CTV to specified margin.

**Table 2:** Volume statistics for all the reference ROIs delineated by radiation oncologist

Categorized organ	Organ name	Volume on CT_1_ (cm^3^)
Large organs (volume ≥ 8 cm^3^)	Left parotid	25.9 ± 8.3
	Right parotid	23.1 ± 5.8
	Spinal cord	28.1 ± 13.2
	Brainstem	20.9 ± 7.5
	Left temporal lobe	66.7 ± 24.3
	Right temporal lobe	70.2 ± 22.1
Small organs (volume < 8 cm^3^)	Left eye lens	0.3 ± 0.1
	Right eye lens	0.4 ± 0.2
	Left optic nerve	0.6 ± 0.5
	Right optic nerve	0.7 ± 0.3
	Left cochlea	1.1 ± 0.9
	Right cochlea	1.0 ± 0.7

All the patients were re‐planned adaptively with prescription dose ranging from 6600 cGy to 7000 cGy depending on the tumor classification by the ROs. Weekly re‐plans were made according to the manually delineated contours by the RO on each corresponding weekly CT scans. All the weekly re‐plans kept the same beam settings and dose constraints with the corresponding primary treatment plan.

### 2.C Deformable image registration algorithms

In this study, two commercial deformable image registration algorithms integrated in RayStation TPS were evaluated. One is a hybrid DIR algorithm called ANACONDA which uses a combination of image intensity information and anatomical information.^27^ The objective is a non‐linear optimization problem which maintains image similarity as well as uses controlling contours for driving the deformation to make the deformation anatomically reasonable. The other is a novel biomechanical DIR algorithm called MORFEUS which computes the displacement field by solving a linear elasticity problem using the finite element method.^28^ The objective function is setup by controlling ROIs represented by meshes and leaves out image gray scale information. Both the two commercial algorithms were set to the same pre‐executed resolution for comparison purpose.

### 2.D Contour propagation and Dose accumulation

**Figure 1:**
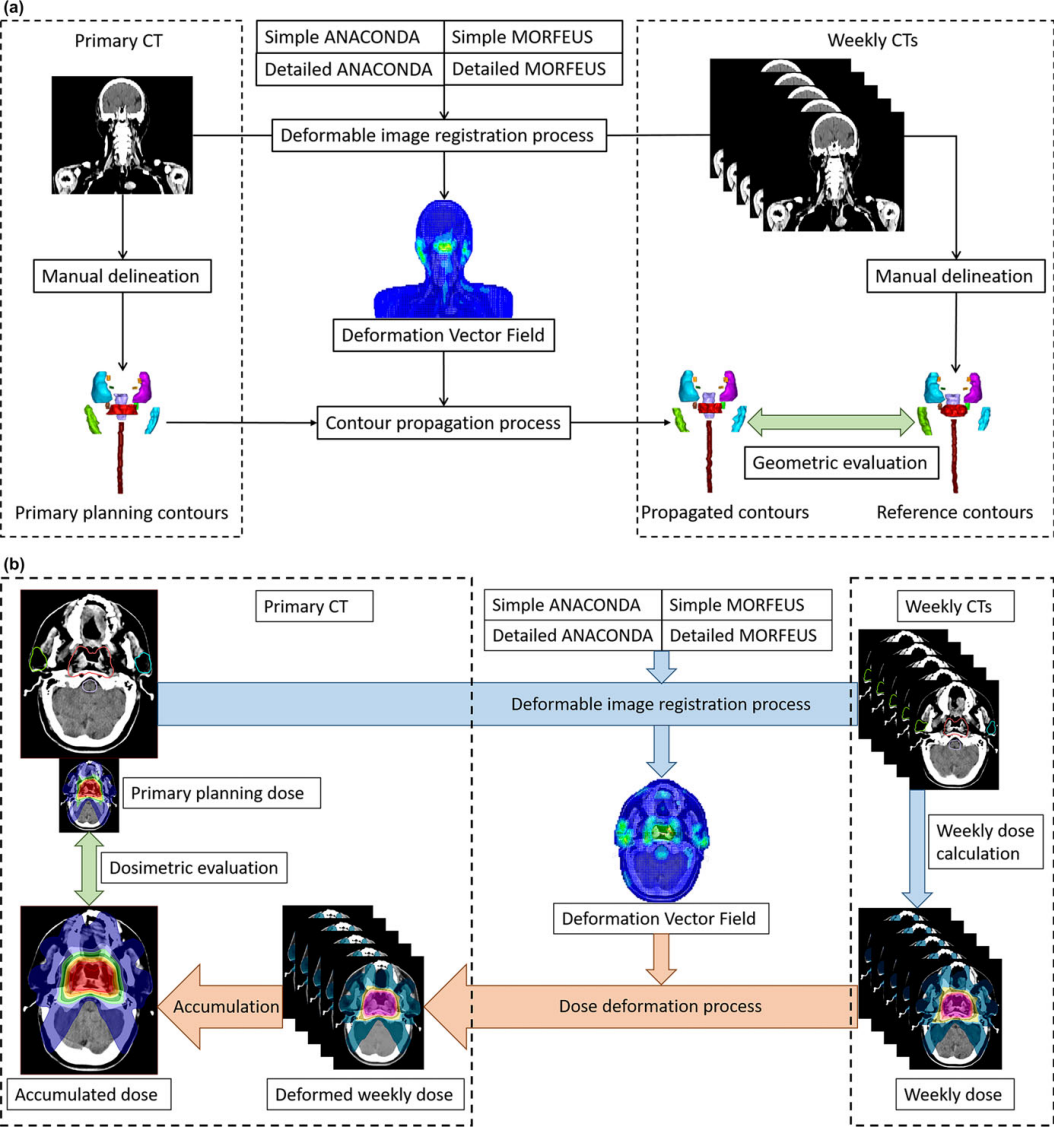
Workflow of the contour propagation and dose accumulation process. (a) Contours were deformably propagated from the primary CT to each weekly CTs using the DVFs generated by different DIRs for geometric evaluation; (b) Weekly doses for each patient were deformed using the DVFs generated by different DIRs and totally accumulated to the primary CT for dosimetric evaluation.

For each patient, the primary CT and weekly CTs both with RO delineated contours were selected for the contour propagation comparison. Though deformable image registration is a relatively complex process, its software interface is relatively simple in the commercial TPS. The quality of DIR processing output depends on both the commercial DIR algorithm itself and the DIR presetting parameters, which will finally influence the contour propagation and dose accumulation results. The DIR presetting parameters adopted in RayStation is the controlling ROI specification which is used as the effective constrains to direct the DIR algorithm to better deformation vector field (DVF). For contour propagation comparison, we used four DVFs to map the RO delineated contours on primary CT to each weekly CT including the GTV and reference OARs. The four different DVFs were generated by the simple, detailed presetting ANACONDA algorithm, and simple, detailed presetting MORFEUS algorithm. The difference between the simple and detailed presetting is the controlling structure. The simple presetting only uses the external ROI while the detailed uses both the external ROI and all the reference ROIs. In summary, on each weekly CT of all the ten patients, five sets of contours were evaluated including four DIR propagated contour sets from the primary CT and one manually delineated reference contour by RO. The workflow of contour propagation was illustrated in Fig. 1(a).

As for the dose accumulation, all the weekly doses were accumulated to the corresponding primary CT deformably based on the four DVFs by the two DIR algorithms with both simple and detailed presetting parameters, as shown in Fig. 1(b).

### 2.E DIR evaluation metrics

#### 2.E.1 Geometric

For geometric comparison, the DICE index was adopted to evaluate the spatial overlap between the volume surrounded by the reference manually delineated and DIR‐propagated contours.^26^ The DICE index is a coefficient to calculate the grade of two volumes’ overlap as follows:

(1) $$\text{DICE index}=2\times \frac{Volume1\cap Volume2}{Volume1+Volume2}$$

where *Volume1* and *Volume2* represent the volumes of selected reference contours acquired by manual delineation and DIR propagation, respectively.^29^ The DICE index has a value ranging from 0.0 to 1.0, with 0.0 meaning non‐overlap and 1.0 meaning totally coincident.

Another geometric evaluation index was the Hausdorff distance (HD) to quantify the max distance of all the nearest points between RO delineated and DIR‐propagated contours, defined as:

(2) $$\text{Hausdorff Distance}=\max\{\min_{a\in A}d\left(a\right),\min_{b\in B}d\left(b\right)\},$$

where $\min_{a\in A}d\left(a\right)$ is the minimum distance of all points on the contour A to points on the contour B, and a represents the point on contour A. The similar definition is used for $\min_{b\in B}d\left(b\right)$.^26^ The DICE index was used to evaluate the overall spatial overlap of two contour volumes, while the Hausdorff distance to quantify the extreme shift of two contours.

#### 2.E.2 Dosimetric

For dose evaluation, all the accumulated weekly doses were deformed and accumulated on primary CT_1_ using different DIR presetting and the accumulated doses were compared with the primary planning dose on CT_1_. For the GTV, the max dose D_max_, min dose D_min_, and mean dose D_mean_ as well as the dose to 95% of the volume D_95_ were evaluated. Also, the Homogeneity Index (HI) value was adopted to analyze the uniformity of the dose distribution in the target volume, defined as:

(3) $$\text{Homogeneity Index}\;\left(\text{HI}\right)=D_{5}/D_{95}$$

where D_5_ is the dose to 5% of the target volume and D_95_ is the dose to 95% of the target volume.^30^ The ideal HI value is 1 and it increases as the plan become less homogeneous. For organs at risk, D_max_ and D_mean_ were used to evaluate the OAR dose. All the accumulated dose variations were counted relative to the primary planning dose as shown in the dosimetric evaluation procedure in Fig. 1(b).

## 3 RESULTS

### 3.A Example patient

**Figure 2:**
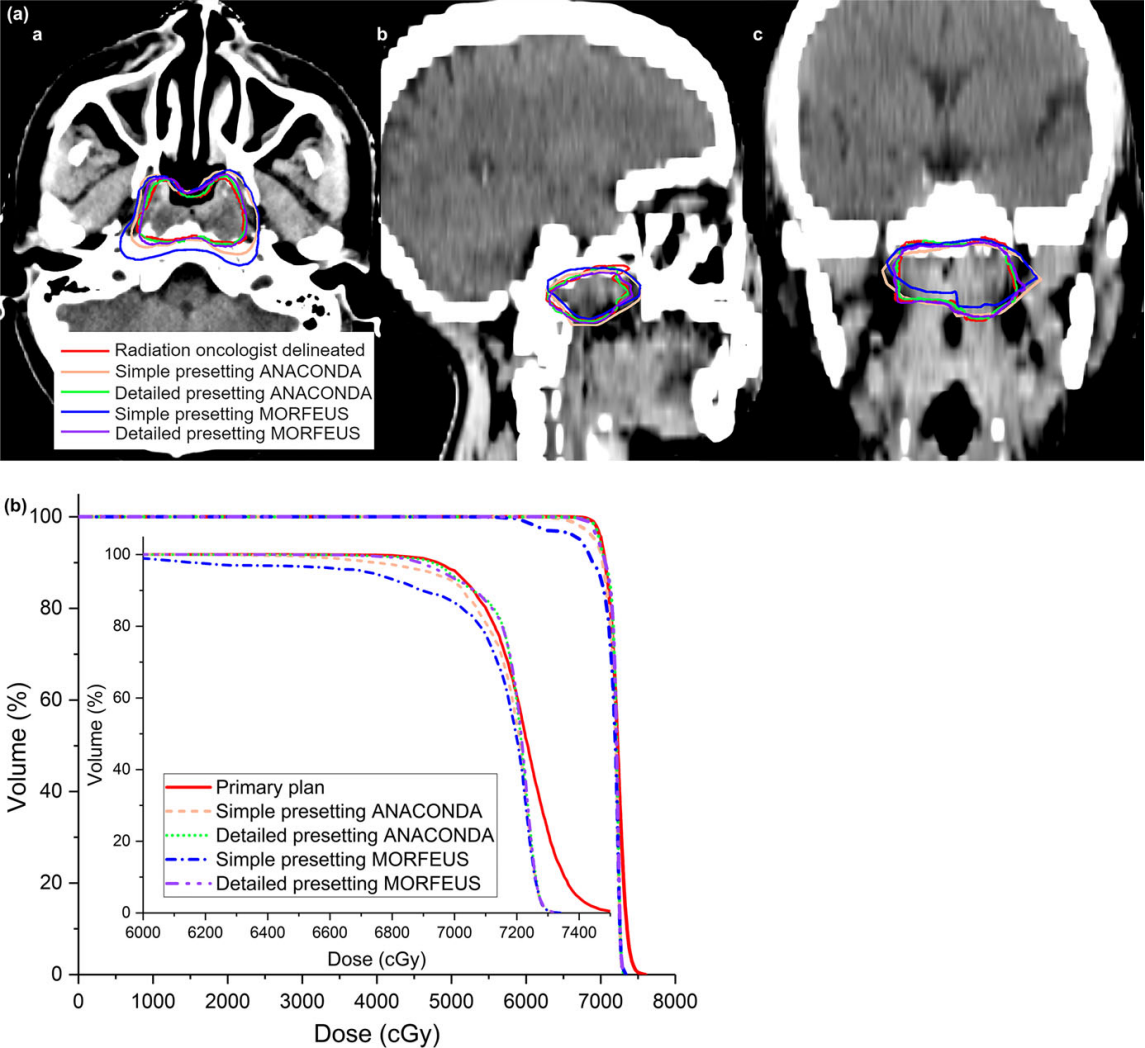
Geomtric and dosimetric variations using different DIR processings for one typical patient. (A). Geometric variations between the RO delineated and the DIR propagated GTV contours on the weekly CT of one typical patient (a) in axial view (b) in sagittal view (c) in coronal view; (B). Comparison of the DVH line for the GTV between the primary planning dose and the DIR‐accumulated weekly doses.

For the GTV contour propagation, the contours mapped by the detailed presetting were found to show better consistency with RO delineated contour, compared to simple presetting for both the ANACONDA and MORFEUS DIR algorithms. Figure 2(A) shows the axial, sagittal and coronal views for one typical patient. This observation also held true for GTV dose accumulation. The DVH of the detailed presetting were found to show better consistency with the primary plan's DVH compared to the simple presetting for both ANACONDA and MORFEUS DIR algorithms, as shown in Fig. 2(B).

### 3.B Geometry

**Figure 3:**
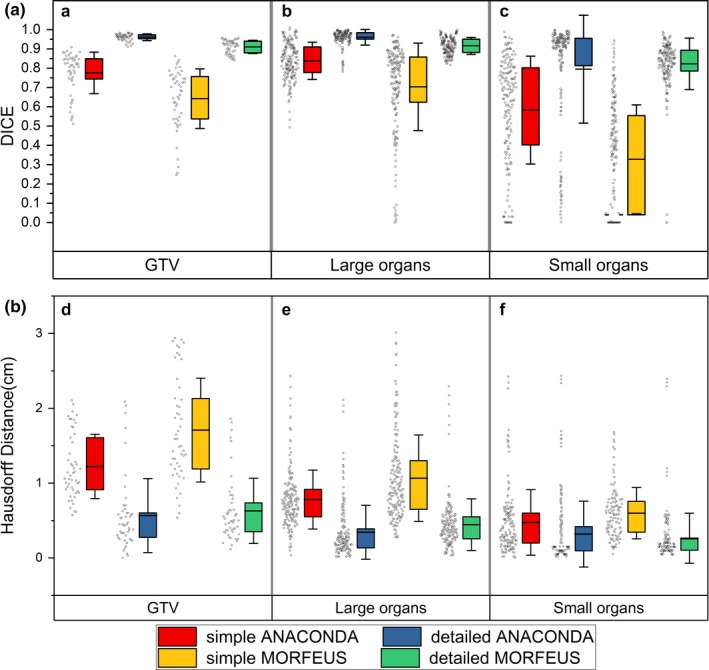
DICE and Hausdorff distance variations of all the patients. (A) Box and whisker plot showing the DIR‐caused DICE coefficient variations for (a) the GTV (b) the large organs (c) the small organs. The limits of each box represent the 25th and 75th percentiles, the whisker represents the standard deviation, and the middle black line represents the average value. The dots next to each box show the trend of the corresponding statistics of all the ten patients; (B) Box and whisker plot showing the DIR‐caused Hausdorff distance variations for (d) the GTV (e) the large organs (f) the small organs. The limits of each box represent the 25th and 75th percentiles, the whisker represents the standard deviation, and the middle black line represents the average value. The dots next to each box show the trend of the corresponding statistics of all the ten patients.

Figure 3(A) shows the DICE statistics of all the ten patients. The DICE coefficient variations of the GTV were found to be 0.78 ± 0.11, 0.96 ± 0.02, 0.64 ± 0.15, and 0.91 ± 0.03 for simple ANACONDA, detailed ANACONDA, simple MORFEUS, and detailed MORFEUS, respectively. For both algorithms, the detailed presettings are better than the simple in terms of the absolute DICE values and the variation. Compared to small organs, the DICE index is higher for large organs under the same DIR condition with the maximum 0.38 variation.

Figure 3(B) depicts the statistics of the HD results. The variations between the two algorithms also become worse when using simple presetting, and reach to maximum in GTV with 0.49 cm. For each algorithm, the detailed presetting leads to better HD results than the simple. The maximum difference between the simple and the detailed presetting is 1.08 cm in GTV using MORFEUS. The HD value is relatively lower in small organs compared to large organs using the same DIR, and the maximum variation shows with 0.46 cm using simple MORFEUS.

### 3.C Dosimetry

**Figure 4:**
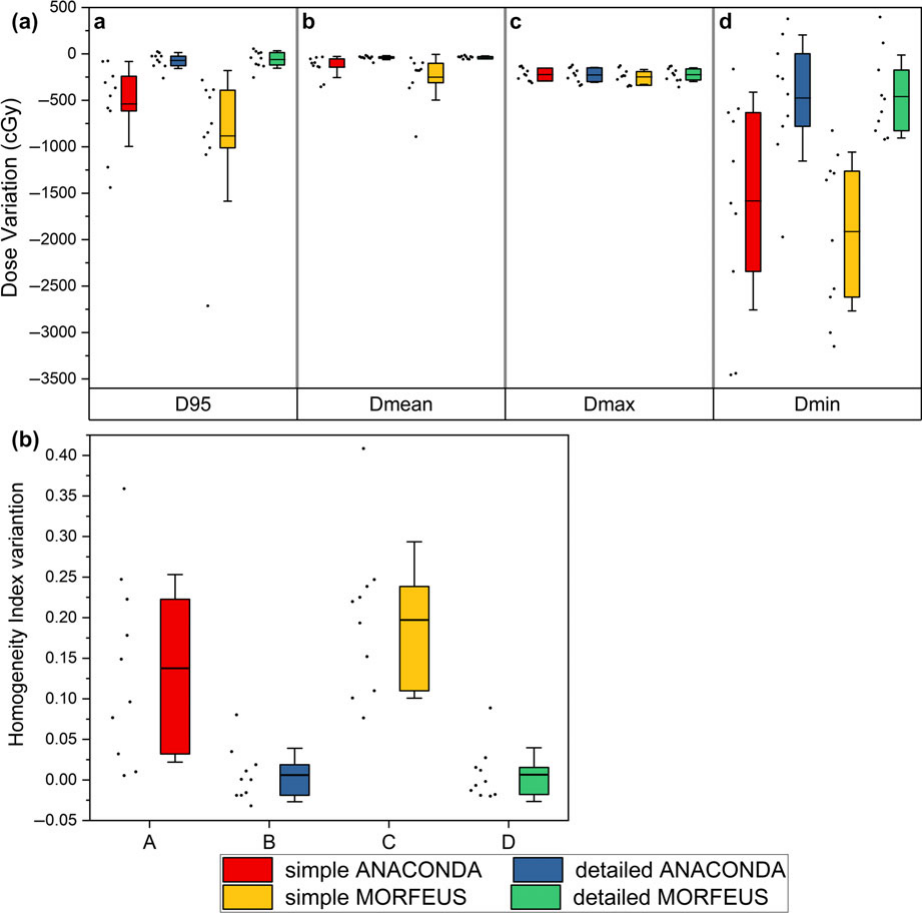
Dosimetric variations of the GTV for all the patients. (A) Box and whisker plot showing the DIR‐caused accumulated dose variations of the GTV for (a) D_95_ (b) D_mean_ (c) D_max_ (d) D_min_. The limits of each box represent the 25th and 75th percentiles, the whisker represents the standard deviation, and the middle black line represents the average value. The dots next to each box show the trend of the corresponding statistics of all the ten patients; (B) Box and whisker plot showing the DIR‐caused accumulated dose variations of the GTV for Homogeneity Index. The limits of each box represent the 25th and 75th percentiles, the whisker represents the standard deviation, and the middle black line represents the average value. The dots next to each box show the trend of the corresponding statistics of all the ten patients.

Figure 4(A) shows the GTV's dosimetric variation statistics of the ten patients between the DIR accumulated and primary planning dose. In general, there are variations between the ANACONDA and MORFEUS algorithms, but again the main differences are between the simple and detailed presettings. The variations are quite large with simple presetting and they are 344.6 cGy, 109.9 cGy, 329.0 cGy for D_95_, D_mean_, D_min_ by average, respectively. On the contrary, these variations are dramatically reduced to be less than 20 cGy with detailed presettings.

Figure 4(B) shows the HI index statistics. For the GTV, the corresponding Homogeneity Index variations were found to be 0.137 ± 0.115, 0.006 ± 0.032, 0.197 ± 0.096, and 0.006 ± 0.033 respectively. Variations exist between the two algorithms and reach to maximum of 0.060 when using simple presetting. For the same algorithm, detailed presetting leads to better HI results than the simple presetting, and the maximum variation is 0.191 between the simple and detailed MORFEUS.

**Figure 5:**
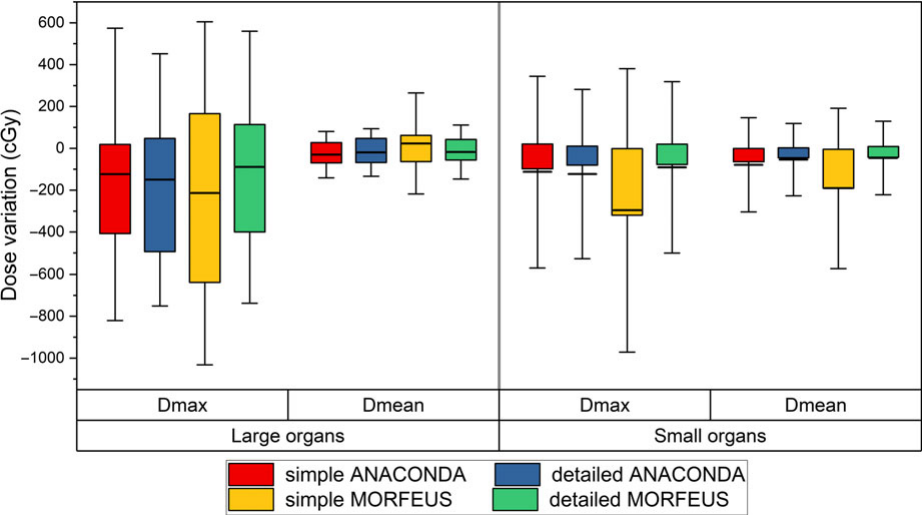
Box and whisker plot showing the DIR‐caused accumulated dose variations of (a) the large organs and (b) the small organs for D_max_ and D_min_. The limits of each box represent the 25th and 75th percentiles, the whisker represents the standard deviation, and the middle black line represents the average value.

Figure 5 shows the large and small organs’ dosimetric variation statistics between the DIR accumulated and primary planning dose. It demonstrates that variations exist between the two algorithms. The variations expand when using simple presetting and reach to maximum with D_max_ of 182.8 cGy and D_mean_ of 111.5 cGy for small organs. For the same algorithm, the detailed presetting leads to better results than the simple. And the maximum variations are shown in small organs with D_max_ 204.4 cGy, D_mean_ 144.0 cGy between the simple and detailed MORFEUS. Compared to small organs, the D_max_ and D_mean_ results are better under the same DIR condition. The variations of D_max_ and D_mean_ reach to maximum with 81.9 cGy and 213.7 cGy between the small and large organs when using simple MORFEUS.

## 4 DISCUSSION

In this study, ten H&N patients with weekly CTs were adopted to evaluate two DIR algorithms integrated in the RayStation ART module, namely, the contour propagation and dose accumulation for adaptive radiotherapy. In a previous study that compared commercial DIR algorithm variations, Nie et al. found that the three commercial DIR algorithms adopted by other ART software were able to achieve DICE coefficients above 0.81 in contour propagation.^31^ Pukala et al. found that the commercial DIR algorithms had a relatively low average geometric registration error between 0.5 mm and 3 mm.^32^ Also, both studies show that, although most of the DIR algorithms could achieve acceptable results for contour propagation, the variations in dose accumulation using different DIR algorithms were found to be more profound. This study investigated more parameters to verify the accuracy of two DIR algorithms used in RayStation. The results show that, under the detailed DIR presetting, the DICE coefficients for both the two algorithms reached 0.8 or higher, and the variations were less than 0.05. The HD values were found to be also consistent with the maximum difference of less than 0.1 cm. And the mean values of the dose variation statistics were lower than 60 cGy, with HI index showing no significant variation. Under the simple presetting, however, the average geometric variations expanded by as much as 40% and the average dosimetric variations expanded by nearly 50% relative to detailed presetting between the two algorithms. Our research confirms the previous research conclusions under detailed presetting but further shows that, when the DIR presetting was simplified in clinical use, the variations between the two DIR algorithms expanded significantly for contour propagation and dose accumulation process.

Deformable image registration is a complex calculation process involving a large number of presetting parameters for research purposes. When it comes to clinical use, however, the presetting parameters will need to be reduced to maintain computational efficiency. The main DIR presetting parameters offered by RayStation is controlling contours that are used for driving the deformation to make the deformation anatomically reasonable. This study explored the influence of such different DIR presettings on contour propagation and dose accumulation in RayStation for the first time. We found that the contour propagation and dose accumulation results obtained by detailed DIR presetting are better than the simplified conditions, as expected. Overall, as shown in the figures, the variations are more significant for different DIR presettings than for different algorithms in clinical use.

This study further explored the influence of ROI's volume on DIR contour propagation and dose accumulation. Previous study by Kumarasiri et al. showed that, under the same DIR conditions, the averaged DICE coefficients for large and small organs contour propagation were 0.82 and 0.59 respectively, and DIR had better performance in large organs compared to small organs for contour propagation process.^26^ As shown by Fig. 3(A), the DICE coefficients are higher for large organs than small organs, and the maximum variation is 0.38 when using the same DIR algorithm with the same presetting. However, the mean value of HD for large organs is slightly higher than that for the small organs, with the maximum variation of 0.46 cm. This is because the DICE coefficient represents the overall volume overlap rate in 3D space and the HD value represents the extreme shift. This result confirms and complements Kumarasiri et al.'s findings.^26^ The dosimetric results in Fig. 5 show that, the variations of D_max_ and D_mean_ for large organs are also lower than small organs under the same DIR conditions. The maximum dosimetric variations were 81.9 cGy for D_max_ and 213.7 cGy for D_mean_ between large and small organs. This result further confirms that, DIR not only performs better for large organs in the overall contour propagation, but also has better performance in dose accumulation compared to small organs.

Overall, the results of this study indicate that RayStation's two DIR algorithms differ in contour propagation and dose accumulation process for H&N adaptive radiotherapy. The dose accumulation process is more complicated than the contour propagation process and can result in more complex variation. And, when simple DIR presetting is adopted, the differences between the two algorithms expand significantly. Therefore, it is necessary to validate the DIR process when more than two DIR algorithms are used together clinically to ensure that the DIR process will not produce excessive deviations between the two algorithms, even under relatively simple DIR presetting. In addition, relative to the variation caused by different algorithms, the clinical DIR presettings can cause a greater degree of deviation for contour propagation and dose accumulation. For the right use of DIR in clinical, a standard usage protocol should be established in each organization to regulate the user's reasonable DIR presettings to ensure the consistency and accuracy of the final result in adaptive radiotherapy. Finally, since the small organs are subjected to significant errors during contour propagation and dose accumulation process using DIR, the users need to pay more attention to the accuracy of the DIR results of the small volume ROIs.

The limitation of this study is that manual delineation was used as the reference contour, thus we can only estimate the influence of above factors to contour propagation and dose accumulation using different DIR. It is not possible in this study to further quantify which algorithm is more accurate, due to the lack of ground truth deformation. Using manual delineation as a reference is clinically easier to achieve intuitive results, and many reports such as Kumarasiri et al.,^26^ Gardner et al.,^33^ and Rigaud et al.^34^ have used this method to evaluate different DIRs. However, considering the necessity of DIR QA, we believe that further research is still necessary to find a clinically feasible method to obtain ground truth deformation for DIR process. At present, the most widely studied methods are to use physical phantoms or computational phantoms to obtain ground truth deformation. But the problem of physical phantoms is that the workload is too great, and it is labor intensive to create a physical phantom to simulate every clinical scenario. In comparison, it is convenient to use the computational phantom instead, but as Nie et al. reported,^31^ the ground truth deformation is actually calculated by computerized algorithms, but the existing nonbiomechanical algorithms do not accurately describe the elastic change of the human tissues and may generate nonphysical deformation. Even biomechanical algorithms may also generate deformation errors due to uncertainties in the Young's Modulus, for different organs. But computational phantom represents a relatively simple and feasible approach. With DIR's growing usage in clinical, especially for adaptive radiotherapy, the clinical DIR QA problem will draw more attention, and the release of AAPM TG‐132 report is expected to provide guidance for further research.^35^

## 5 CONCLUSION

In this study, we evaluated the contour propagation and dose accumulation variations induced by the DIR process for ten H&N adaptive radiation therapy cases retrospectively using two integrated DIR algorithms in RayStation. The results showed that there were significant variations in the DICE coefficients, the Hausdorff distance between the two algorithms, especially under simple presetting condition. The dosimetric results lead to the same conclusion. DIR presettings have been found to have more significant influence on the final results, and the detailed presetting showed less significant variation in contour propagation and dose accumulation than the simple presetting. Compared to large ROIs, small ROIs were easier to produce more significant variation in both the contour propagation and dose accumulation. As more treatment planning systems integrate the DIR module, it's necessary for each organization to establish their DIR protocols and quality assurance procedures for adaptive radiation therapy.

## CONFLICT OF INTEREST

The authors declare no conflict of interest.
